# Upregulation of heat shock factor 1 transcription activity is associated with hepatocellular carcinoma progression

**DOI:** 10.3892/mmr.2014.2547

**Published:** 2014-09-08

**Authors:** SHULIAN LI, WANLI MA, TENG FEI, QIANG LOU, YAQIN ZHANG, XIUKUN CUI, XIAOMING QIN, JUN ZHANG, GUANGCHAO LIU, ZHENG DONG, YUANFANG MA, ZHENGSHUN SONG, YANZHONG HU

**Affiliations:** 1 State Key Laboratory of Antibody Engineering, Department of Genetics and Cell Biology, Henan University School of Medicine, Kaifeng, Henan 475004, P.R. China; 2 Department of Surgery, Huaihe Hospital Affiliated to Henan University, Kaifeng, Henan 475004, P.R. China; 3 Department of Surgery, Shanghai Tenth People’s Hospital Affiliated to Tongji University, Shanghai 20072, P.R. China; 4 Department of Anatomy and Cell Biology, Georgia Regents University, Augusta, GA 30912, USA

**Keywords:** heat shock factor 1, retinoblastoma protein, hepatocellular carcinoma, phosphorylation

## Abstract

Heat shock factor 1 (HSF1) is associated with tissue-specific tumorigenesis in a number of mouse models, and has been used a as prognostic marker of cancer types, including breast and prostatic cancer. However, its role in human hepatocellular carcinoma (HCC) is not well understood. Using immunoblotting and immunohistochemical staining, it was identified that HSF1 and its serine (S) 326 phosphorylation, a biomarker of HSF1 activation, are significantly upregulated in human HCC tissues and HCC cell lines compared with their normal counterparts. Cohort analyses indicated that upregulation of the expression of HSF1 and its phospho-S326 is significantly correlated with HCC progression, invasion and patient survival prognosis (P<0.001); however, not in the presence of a hepatitis B virus infection and the expression of alpha-fetoprotein and carcinoembryonic antigen. Knockdown of *HSF1* with shRNA induced the protein expression of tumor suppressor retinoblastoma protein, resulting in attenuated plc/prf5 cell growth and colony formation *in vitro*. Taken together, these data markedly support that HSF1 is a potential prognostic marker and therapeutic target for the treatment of HCC.

## Introduction

Heat shock factor 1 (HSF1) is a member of heat shock transcription factors that are able to differentially regulate the expression of heat shock proteins in response to a variety of stresses (1,2). These heat shock proteins function as molecular chaperones, regulating cellular homeostasis via modulating protein folding, assortment, stability and protein-protein interactions. This adaptive process is known as the heat shock response (1). HSF1 is the predominant heat shock response transcription factor. In addition to mediating the heat shock response, it is associated with numerous other cellular processes, including tissue development (e.g. brain, testis and placenta) (3), inflammation and tumorigenesis (4). In humans, HSF1 and its associated heat shock proteins are upregulated in the majority of tumor tissue types (e.g. lymphoma, lung, breast and prostate cancer) (5–7), and are involved in regulating tumor cell hyperproliferation, metabolism, metastasis and chemotherapy resistance (8–11). Certain heat shock proteins (e.g. HSP90 and HSP70) have been targeted for cancer therapy and the creation of tumor prognostic biomarkers (12–14). In animal models, HSF1 is important for p53 mutation-induced lymphoma (15), diethylnitrosamine (DEN)-induced hepatocellular carcinoma (16), dimethylbenz(a)anthracene (DMBA)-tetradecanoylphorbol acetate (TPA)-induced skin cancer (4) and human epidermal growth factor receptor 2 (Her2)-induced breast cancer (5,9). In these tumor models, HSF1 is involved in regulating tumor initiation, development and metastasis, and is considered a novel non-oncogenic oncogene. These data suggested that HSF1 may act as a novel candidate for the development of new cancer prognostic biomarkers.

The biomarkers that represent HSF1 transcription activation in tumor tissues are currently being investigated. Protein post-translational modifications, e.g. phosphorylation, have important roles during HSF1 activation. Under physiological conditions, HSF1 forms monomers or heterodimers with an HSP90-HSP70 chaperone complex without transcriptional activity (17,18) and is constitutively phosphorylated at serine (S)303 and S307 (19,20). Upon heat shock, HSF1 dissociates from the HSF1-HSP90-HSP70 chaperone complex and becomes activated upon hyperphosphorylation at S230 and S326 (21–23). Mutations of HSF1/S326 disrupt its transcriptional activity under heat shock conditions, referring the phosphorylation of S326 as a biomarker of HSF1 activation (23). Recently, Mendillo *et al* (7) reported that hyperphosphorylation of HSF1/S326, which is upregulated in breast cancer compared with the normal counterparts, was used as a biomarker to indicate HSF1 activation in breast cancer. The constitutive activation of HSF1 in breast cancer contributes to the expression of a group of malignant program genes in addition to the heat shock proteins and this HSF1-regulated malignant program was also active in colon and lung cancer (7).

Hepatocellular carcinoma is the fifth most common, with the third highest mortality rate of all cancer types worldwide. It dominantly occurs in Asian countries, including China, Japan and Southeast Asian countries (24). HCC is closely correlated with the infection of hepatitis B virus (HBV), HCV, aspergillus flavus infections, as well as cirrhosis and obesity (25). A number of proteins have been identified as biomarkers for HCC diagnosis and prognosis, including alpha-fetoprotein (AFP), AFPLens culinaris agglutinin-reactive AFP, des-gamma-carboxy prothrombin, glypican-3, osteopontin, and others, including squamous cell carcinoma antigen-immunoglobulin M complexes, alpha-1-fucosidase, chromogranin A, human hepatocyte growth factor and insulin-like growth factor (26). However, none of these biomarkers are efficacious for the early diagnosis of HCC, and therefore, further studies are required to identify novel specific biomarkers of HCC to improve the prognosis. The accumulative evidence indicates that HSF1 and its downstream HSPs are upregulated in HCC tissues. Knockdown HSP70 and HSF1 triggered apoptosis of an HCC cell line *in vitro* (27) and the inhibition of DEN-induced HCC *in vivo* (16). HSP27, which is upregulated in HCC tissues, is also elevated in HCC patient serum and is correlated with HCC prognosis (28). It was reported that HSF1 is upregulated in prostate cancer and HCC (27). However, the possible role of HSF1 as a prognostic marker of HCC has not been well studied.

The present study investigated HSF1 protein expression and its phosphorylaton at S326 in HCC tumor tissues and HCC cell lines. Knockdown of HSF1 in the HCC cell line plc/pfr5 was achieved with small hairpin (sh)RNA, and its effects on protein expression, cell growth and colony formation were assessed. It was explored whether of HSF1 and phospho-S326 may be used as biomarkers of HCC progression and as potential candidate targets for HCC therapeutics.

## Materials and methods

### Cell culture and plasmids

The cell lines SMMC7042 (Chinese Academy of Sciences, Shanghai, China), HepG2 (ATCC, Manassas, VA, USA), plc/prf5 (ATCC), SM7721 (Chinese Academy of Sciences) and Chang liver cells (China Military Medical Science Academy, Beijing, China) were routinely grown in Dulbecco’s modified Eagle’s medium (DMEM) containing 10% FBS and 100 μg/ml ampicillin-streptomycin mixture in a 37°C incubator with 5% CO_2_. The cells were passaged every two days. The pLTHR-shRNA-HSF1 plasmid was used as a retroviral vector expressing the shRNA targeting the human HSF1 sequence CAG GAG CAG CTC CTT GAG A (29). The pLTHR-shRNA-enhanced green fluorescence protein (EGFP) was used as the scrambled shRNA.

### Recombinant retrovirus expresses shRNA-HSF1

The pLTHR-shRNA-HSF1 and pLTHR-scramble constructs were transiently transfected into 293 amph cells for retrovirus packaging. The cell supernatants, which were collected and mixed with 2 μg/ml polybrane, were used to infect the plc/prf5 cells for 12 h. Following selection with 2 μg/ml of puromycin for three days, the live cells were pooled and used for the experiments (e.g. immunoblotting, cell growth and colony formation assay and cell cycle analysis).

### Immunohistochemical staining

Primary HCC tissues were kindly provided by Dr. Song Zhenshun (Department of Surgery, Shanghai Tenth Hospital Affiliated to Tongji University, Shanghai, China) were imbedded in paraffin and selected for immunohistochemical staining using the standard method. Briefly, following deparaffinization, rehydration and antigen retrieval, the slides were blotted in 3% bovine serum albumin/phosphate-buffered saline buffer for 1 h and incubated with primary rabbit polyclonal antibody against Hsf1 for 1–2 h. Following washing out unbound primary antibody, the slides were then incubated with secondary antibody conjugated with alkaline phosphatase (AP). The slides were developed in DAB buffer and counterstained with hematoxylin. The slides were photographed with the ZEISS 540 microscope under 40x-index (Carl Zeiss, Jena, Germany). The study was approved by the Ethics Committee of Shanghai Tenth Hospital Affiliated to Tongji University.

### Immunoblotting, immunoprecipitation and glutathione S-transferase (GST)-pull down

The cells were lysed in modified radioimmunoprecipitation assay buffer [50 mM Tris-Cl, pH 7.4, 150 mM NaCl, 0.25% deoxycholate, 0.5% NP-40, 1× protein inhibitor cocktail and 1× phosphatase inhibitor cocktail (Sigma, St. Louis, MO, USA)]. The procedures for immunoblotting, immunoprecipitation and *in vivo* GST-pull down were performed as described previously (Zhang *et al* (30), 2010). The rabbit polyclonal antibodies against HSF1 and HSP70 were purchased from Invitrogen Life Technologies (Carlsbad, CA, USA). The rabbit polyclonal anti-phospho-Hsf1/S326 antibody was purchased from Enzo Life Sciences, Inc., Famingdale, NY, USA). The antibodies against pRB and p53 were obtained from Santa Cruz Biotechnology, Inc. (Santa Cruz, CA, USA).

### MTT assay, colony formation and cell cycle analysis

For the MTT assay, 2×10^3^ plc/prf5 cells that stably expressed shRNA-*HSF1* and plc/prf5-scramble were seeded into 96-well plates and grown for up to seven days. Every day, MTT reagent was added to the media 4 h prior to cell collection. The MTT-labeled cells were homogenized in lysis buffer containing 0.1% NP-40/isopropanol for 10 min. The optical density (OD) value was calculated at an absorbance wavelength of 599 nm. For colony formation, 500 cells were seeded into 60-mm plates and grown for seven days. The cells were stained with 0.1% crystal violet for 30 min. The data expressed represent three independent experiments. For cell cycle analysis, equal numbers of the cells expressing shRNA-*HSF1* or plc/prf5-scramble were cultured for 24 h. The cells were then collected and fixed in 70% ethanol. Following propidium iodide (PI) staining, the cell cycles were analyzed by flow cytometry (BD FACSCalibur, San Jose, CA, USA).

### Statistical analysis

The χ^2^-test and Spearman’s rho analysis using SPSS software (SPSS, Inc., Chicago, IL, USA), and Student’s t-test using Quantity One software were applied for statistical analysis. P<0.05 was considered to indicate a statistically significant difference.

## Results

### HSF1 protein and phospho-S326/HSF1 are upregulated in HCC cell lines and tissues

**Figure 1:**
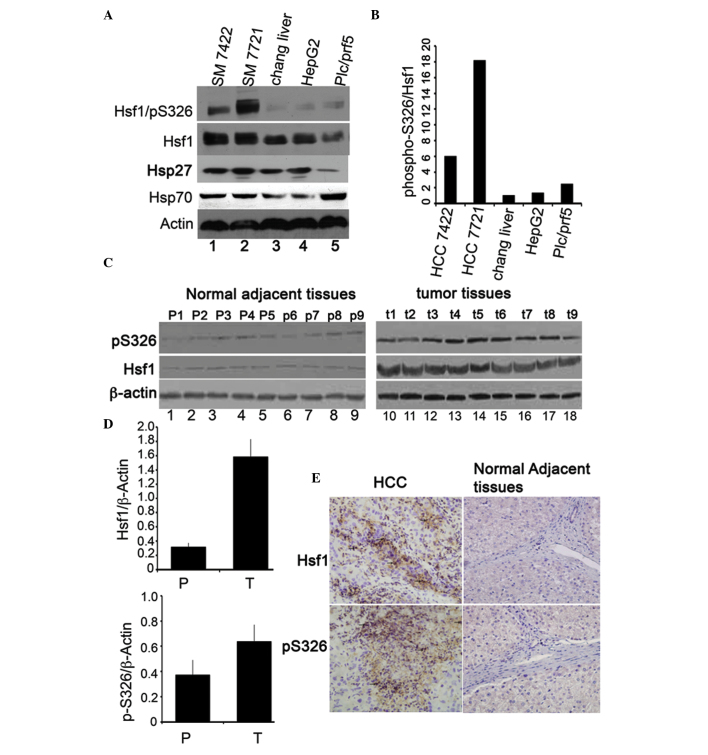
Protein expression of HSF1 and phosphorylation of HSF1/S326 in HCC cell lines and tissues. (A) Immunoblotting of HSF1, phospho-S326/HSF1 and β-actin in four HCC cell lines: Lane 1, SM7024; lane 2, SM7721; lane 3, immortalized Chang liver cell line; lane 4, HepG2; lane 5, Plc/prf/5. (B) The percentage of phospho-S326/HSF1 was determined by normalization of the density of phosphorylation of HSF1/S326 to the density of the HSF1 protein. (C) Immunoblotting of HSF1 and phospho-S326/HSF1 in HCC tissues and the normal counterparts. N1–N9 denote nine cases of normal counterpart tissues (left panel) and T1–T9 represent nine cases of HCC tissues (right panel). (D) Quantity of the Hsf1 proteins (upper panel) and phospho-S326 (lower panel). (E) Immunohistochemical staining of the expression of HSF1 and phospho-S326/HSF1 in HCC tissue (left panels) and in the normal counterparts. Magnification, ×40. ^*^P<0.05, the expression levels of Hsf1 and phospho-Hsf1/S326 in normal adjacent tissues compared with that in HCC tissues. HSF1, heat shock factor 1; HCC, hepatocellular carcinoma.

To further elucidate the activity of HSF1 in human HCC, the expression levels of HSF1 and phospho-S326/HSF1 in the four HCC cell lines and in the immortalized hepatocyte Chang liver cells were assessed by immunoblotting. As indicated in Fig. 1A and B, HSF1 is upregulated in the four HCC cell lines when compared with the immortalized Chang liver cells, but the expression levels of HSF1 vary between the four HCC cell lines (lanes 1, 2, 4 and 5). The expression levels of HSF1 in SM7721 and M7024 was higher than those in HepG2 and plc/prf5 cells. HepG2 and plc/prf5 are two highly differentiated HCC cell lines (31). Consistent with HSF1, the phosphorylation of HSF1/S326 is also upregulated in the HCC cell lines compared with the Chang liver cells (Fig. 1A, upper panel). The phosphorylation ratio of S326 was increased six-fold in SM7024, 18-fold in SM7721, 1.3-fold in HepG2 and 2.5-fold in the plc/prf5 cells (Fig. 1B) compared with that in Chang liver cells. To determine whether levels of HSF1 expression and phospho-S326/HSF1 were upregulated in HCC tissues, nine primary human HCC tissues and their corresponding adjacent normal tissues were assessed by immunoblotting. HSF1 and phospho-S326/HSF1 were upregulated in the HCC tissues compared with their parental adjacent normal tissues (Fig. 1C and D). Consistently, immunohistochemical staining indicated that the HSF1 protein and its phopho-S326 derivative were upregulated in the HCC tissues compared with their adjacent normal tissues (Fig. 1E). These results demonstrated that HSF1 protein expression and transcriptional activity were upregulated in HCC tissues.

### HSF1 expression is correlated with HCC progression

**Figure 2:**
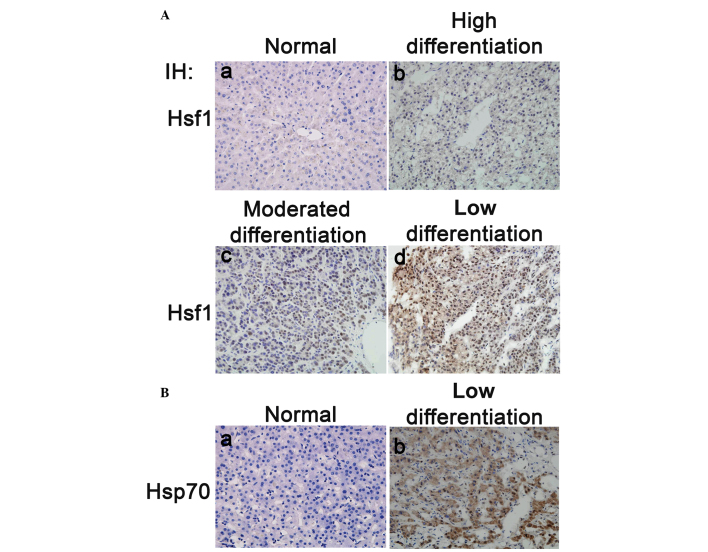
Expression of HSF1 and HSP70 in the diversely differentiated HCC tissues. (A) IH analysis of HSF1 in (a) normal liver tissues and in (b) highly, (c) moderately and (d) poorly differentiated HCC tissues. (B) Expression of HSP70 in (a) normal liver tissues and (b) poorly differentiated HCC tissues. Magnification, ×40. HSF1, heat shock factor 1; HSP70, heat shock protein 70; HCC, hepatocellular carcinoma; IH, immunohistochemistry.

The immunoblotting results demonstrated that both HSF1 protein expression and phosphorylation of S326 were significantly upregulated in the HCC tissues compared with their adjacent normal counterparts. It was therefore hypothesized that HSF1 may act as an effective prognostic marker of HCC. To prove this hypothesis, 67 HCC tissues from HCC patients (who had not received any prior chemotherapy and/or radiotherapy) and 21 normal liver tissues were used to examine the expression of HSF1 by immunohistochemical staining. The results indicated that the expression levels of HSF1 in the moderately and poorly differentiated HCC tissues were notably higher than those in the highly differentiated HCC and normal liver tissues (Fig. 2A). Statistical analysis of cohort studies indicated that 68.7% (n=46/67) of the HCC patients were HSF1-positive compared with 28.6% (n=6/21) of the normal liver biopsies (χ^2^=10.628, P=0.001; Table I), and HSF1 protein levels were significantly increased in HCC tissues. Furthermore, the correlation between the HSF1 expression and HCC malignancies (including HCC metastasis, cancer cell differentiation, early phase HCC and late phase HCC, aging, gender and HBV infection) was studied. Of the 67 HCC patients, 14 out of 27 HCC patients who had intact tumor membranes were HSF1-positive (51.9%). By contrast, 32 out of 40 (80.0%) HCC patients with broken tumor membranes were HSF1-positive and HSF1 expression levels were notably higher in membrane-broken HCC than those in membrane-intact tumors. The χ^2^ analysis results indicated that the expression of HSF1 was correlated with HCC invasion and metastasis (χ^2^=9.76; P=0.015). According to the tumor differentiation characteristics, there were 26, 31 and 10 patients out of the total 67 patients who were diagnosed as poorly, moderately and well-differentiated HCC, respectively. The immunohistochemistry results demonstrated that 96.2% (n=25/26) of the poorly differentiated HCC, 61.3% (n=19/31) of the moderately differentiated HCC and 20% (n=2/10) of the well-differentiated HCC patients were HSF1-positive. Statistical analysis indicated that HSF1 expression was significantly different between the poorly and moderately differentiated HCC, and between the moderately and well-differentiated HCC samples (χ^2^=5.159; P<0.05). This suggested that HSF1 expression was closely associated with malignant HCC progression. Based on the clinical diagnosis [using the tumor, nodes and metastasis grading system], 44.0% of phase I-II HCC tissues (n=11/25) demonstrated low levels of HSF1 protein expression, which was significantly different to the 83.3% of phase III and IV HCC tissues that exhibited high HSF1 expression. However, the expression of HSF1 was not correlated to HCC patient age, gender, HBV infection status, AFP expression levels, Ceacam1 expression levels and portal vein thrombosis (Table I).

To determine whether the expression of HSP70 is correlated with HSF1 in HCC, 40 poorly differentiated HCC tissues were immunohistochemically stained with an HSP70 antibody. Similar to HSF1, HSP70 protein expression was significantly upregulated in the poorly differentiated HCC tissues compared with the non-cancerous tissues (Fig. 2B). The correlation between HSF1 and HSP70 in HCC was studied in a cohort of 40 HCC tissues. A total of 22 HCC samples were both HSF1- and HSP70-positive, 10 samples were both negative for HSF1 and HSP70, 3 HCC samples were HSF1-postive but HSP70-negative, and 5 samples were HSF1-negative but HSP70-positive. The Spearman test results indicated that HSF1 expression was significantly correlated with HSP70 expression (Table II). Taken together, these results strongly supported that the expression of HSF1 is closely correlated with HCC progression and HSP70 is one of the downstream targets of HSF1 in HCC tissues.

### HSF1 knockdown inhibits plc/prf5 cell proliferation

**Figure 3:**
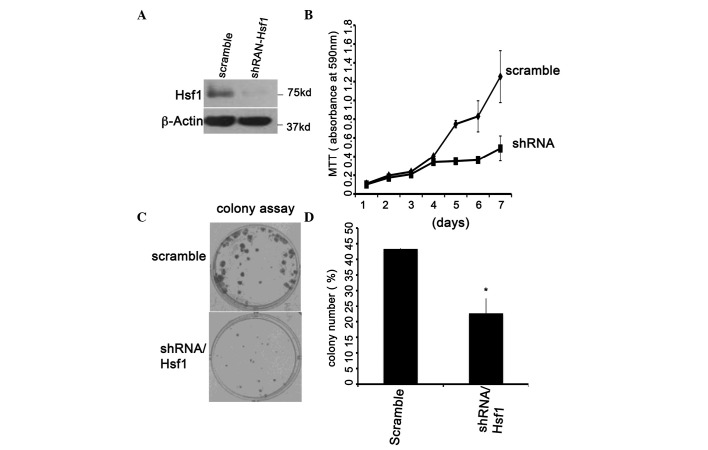
HSF1 regulates plc/prf5 HCC cell growth and colony formation. (A) Immunoblotting of the expression of HSF1 in plc/prf5 cells expressing shRNA against HSF1 (lane 1) and scramble shRNA (lane 2). (B) Knockdown of HSF1 inhibited plc/prf5 cell growth. The growth curve of plc/prf5 cells that stably express shRNA-HSF1 (solid square) or scrambled shRNA (solid diamond) were determined with an MTT assay. (C) Colony formation of the plc/prf5 cells expressing shRNA or scrambled shRNA in the cultured plates. (D) Colonial formation efficiency, which was calculated by dividing the colony numbers by cell numbers. Data were obtained from three independent experiments. For colony formation assay, the 10^3^ cells were seeded in 6-well plates and cultured for 7 days. The colonies were stained with crystal violet solution. The colony numbers divided by the number of initially seeded cells were accounted for. ^*^P<0.01. HSF1, heat shock factor 1; HSP70, heat shock protein 70; HCC, hepatocellular carcinoma; shRNA, small hairpin RNA.

The close correlation between HSF1 and HCC progression in Table I suggested that HSF1 may be a novel therapeutic target of HCC. To determine its therapeutic roles, the plc/prf5 cells were selected for further study as manipulating them for transient transfection *in vitro* is a simple process. The scrambled shRNA and shRNA against HSF1 were transiently transfected into plc/prf5 cells. HSF1 was significantly downregulated by shRNA-*HSF1* but not by the scrambled shRNA (Fig. 3A, lanes 1 and 2). Knockdown of HSF1 expression may significantly inhibit prc/prf5 cell proliferation and colony formation (Fig. 3B and C). These data were consistent with observations reported for other cell types (9) and demonstrated that HSF1 may be a target for HCC therapy.

### HSF1 knockdown induces pRB protein expression

**Figure 4:**
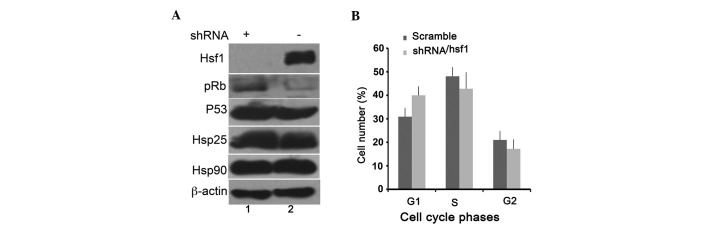
Knockdown of Hsf1 arrests the cells at G1 phase by upregulating the expression of pRB. (A) Immunobotting of the expression of HSF1, pRB, p53, HSP27, HSP90 and β-actin in the plc/prf5 cells containing shRNA-HSF1 (lane 1) and scrambled shRNA (lane 2). (B) Cell cycle analysis of plc/prf5 cells containing shRNA-HSF1 (black bar) or scrambled shRNA (gray bar). ^*^P<0.05, scramble shRNA vs. shRNA-Hsf1. HSF1, heat shock factor 1; HSP, heat shock protein; HCC, hepatocellular carcinoma; pRB, retinoblastoma protein; shRNA, small hairpin RNA.

It has been previously reported that a depletion of HSF1 expression may induce the expression of p53 in E1A-immortalized mouse embryonic fibroblast (MEF) cells, resulting in cell growth inhibition (32). To investigate whether the induction of p53 was also involved in the slow growth of HSF1-knockdown prc/plf5 cells, the cells expressing shRNA-*HSF1* or scrambled shRNA were subjected to immunoblot analysis. The results shown in Fig. 4A showed no difference in p53, HSP90 and HSP27 expression levels between the cells expressing shRNA-*HSF1* and those expressing scrambled shRNA. However, pRB, another tumor suppressor, was evidently overexpressed in the shRNA-*HSF1*-expressing cells compared with the cells expressing scrambled shRNA (Fig. 4A, lanes 2 and 1). Cell cycle analysis indicated that 42% of the shRNA-transfected cells were in G1 phase, as compared with 35% of scrambled cells in G1 phase. There was no statistically significant difference in the number of cells in S and G2 phase between the two cell lines. These results indicated that knockdown of HSF1 may arrest the cell cycle in G1 phase (Fig. 4B) by upregulating pRB protein expression.

## Discussion

Identification of the proteins that are specifically expressed in tumor tissues has been used for targeted tumor therapy and prognosis (33). The present study provided evidence to support the hypothesis that HSF1 may be used as a prognostic biomarker and therapeutic target for HCC. The data demonstrated that HSF1 protein expression and its phospho-S326/HSF1 were significantly upregulated in HCC tissues compared with the adjacent normal tissues. Statistical analysis demonstrated that HSF1 expression and transcriptional activities were closely correlated with HCC development and invasion. Knockdown of HSF1 inhibited prc/plf5 cell growth and colony formation, induced the expression of pRB protein and arrested the cell cycle at G1 phase. The results demonstrated that HSF1 may be a novel prognostic marker and therapeutic target for HCC.

An association of HSF1 with cancer initiation and development has been found in animal models and human cancer tissues (4,9). Knockdown of HSF1 may inhibit mutant p53-induced lymphoma; Her2 induced breast cancer and DEN induced hepatocellular carcinoma in mouse models (4,16). Defective HSF1 slowed down SV40-Tag- (unpublished data) and E1A-induced MEF cell transformation *in vitro* (32). These data strongly suggested that HSF1 activation is closely associated with cell transformation. In addition, knockdown of HSF1 with siRNA may induce human tumor cell apoptosis *in vitro* (4), which supports the evidence that HSF1 has important roles in maintaining tumor development. HSF1 has been found to be associated with several oncogenic pathways. For example, both HSF1 and HSF2 were identified to regulate p53 protein stability by controlling the expression of the proteasome subunits Psmb5 and gankyrin (34). p53 protein is upregulated in E1A-immortalized HSF1^−/−^ MEF cells compared with its parental tissues (32). HSF1 is able to associate with cell cycle regulators cdc20 and polo-like protein kinase 1, participating in the regulation of tumor cell chromosomal stability (35). Furthermore, it is able to modulate the metabolism of glucose and lipids by indirectly regulating insulin receptor protein expression, which has been demonstrated to be important for tumor initiation and development (16). HSF1 was reported to regulate interleukin (IL)-6 expression by binding to and triggering the demethylation of the IL-6 promoter, the latter of which was found to be the key inflammatory factor involved in chronicle inflammation-induced cell transformation (36,37). In breast cancer, HSF1 knockdown inhibited Erb2-induced breast tissue tumorigenesis and tumor metastasis in mouse models (5,9). Deletion of HSF1 was identified to interfere with the HSP90-Her2 complex association, induce p21 protein accumulation and reduce survivin expression (5). By immunohistochemistry and immunoblotting it was identified in the present study that the expression of HSF1 was highly correlated with HCC development and prognosis. HSF1 expression was significantly upregulated in the poorly differentiated, membrane-broken HCC, rather than the normal and highly differentiated HCC tissues (Fig. 2 and Table II). These data provided further supporting evidence that HSF1 is closely associated with HCC development and prognosis, which is consistent with the results of previous studies (27). To determine the underlying signaling pathways involved, HSF1 expression was silenced with shRNA *in vitro* in the HCC cell line plc/prf5. HSF1 knockdown induced significant upregulation of pRB expression, suggesting that in plc/prf5 cells, HSF1 is associated with tumor suppressor pRB instead of p53, which may explain why deletion of HSF1 causes cell growth arrest (Fig. 4B). HSF1 has been reported to modulate p53 protein stability by regulating the expression of 26S proteasome subunits Psmb5 and gankyrin (34). It also directly binds to the promoter, initiating the transcription of target genes, including IL-6 and HSPs. However, it remains elusive how HSF1 negatively regulates pRB expression.

Furthermore, it remains elusive how HSF1 is activated in tumorigenesis. HSF1 is activated by heat shock and other stresses, and the activation of HSF1 is a complex process, involving HSF1 homotrimerization, hyperphosphorylation, acetylation, sumoylation and nuclear translocation. It has been reported that phosphorylation of S230 and/or S326 is an important process for HSF1 activation (22,23). Mutation of S326 and S230 impairs HSF1′s transcriptional activity under heat shock conditions. Ca^2+^/calmodulin-dependent protein kinase II (CAMKII), c-Jun N-terminal kinase (JNK) 1/2 and mammalian target of rapamycin (mTOR) were found to be able to phosphorylate these two sites in response to different stresses (22,23,35). CAMKII is activated by heat shock and glutamine and mediates the phosphorylation of HSF1/S230 (38). JNK1-mediated phosphorylation of HSF1/S320 participates in the regulation of IL-6 expression during the inflammation-induced cell transformation and malignant suspension (36). mTOR is able to sensitize the protoxic signals or nutrient signals and participates in the phosphorylation of S326. Knockdown of mTOR blocks S326 phosphorylation and inhibits the activation of HSF1 in heat shock conditions. These results demonstrate that the phosphorylation of S326 serves as a hallmark for HSF1 activation under heat shock stress. Mendillo *et al* (7) reported that phosphorylation of S326 was detected in breast cancer tissues but not in immortalized breast epithelial cells and normal breast tissues, suggesting that phosphorylation of S326 may be a marker of active HSF1 in tumor tissues (7). According to this evidence, in the present study, the expression of HSF1 and phospho-S326/HSF1 was compared between HCC tissues and adjacent normal tissues. The results demonstrated that the expression of HSF1 protein and phosphorylation of S326 were significantly elevated in HCC tissues compared with the adjacent normal tissues (Fig. 1A and C). The results indicated that HSF1 was activated in HCC tissues. However, the biological effects of HSF1 in HCC remain elusive. Generally, it is noted that the dominant role of HSF1 is to regulate the HSPs chaperone expression, which is important for tumorigenesis. However, Mendillo *et al’*s recent study demonstrated that heat shock and tumorigenesis-activated HSF1 are involved in different signaling pathways (7). Heat shock-activated HSF1 is mainly responsive to the expression of heat shock proteins, while activation of HSF1 in tumor tissues is predominantly responsible for controlling the expression of non-heat shock proteins, e.g. CKS2, LY6K, RBM23, CCT6A, CKS1B, ST13 and EIF4A2. In transformed breast epithelial cells, active HSF1 prefers to bind to the promoters of non-heat shock proteins. By contrast, HSF1 is facilitated to recognize HSPs (HSP70 and HSP90) under heat shock conditions. The distinctive promoter-binding preferences of HSF1 are hypothesized to be regulated by post-translational modifications, including phosphorylation, acetylation, sumoylation and glycosylation.

Clinically, well-established cancer biomarkers are used as diagnostic and prognostic indicators. For example, prostate-specific antigen (PSA) is used for prostate cancer, Erb2 and estrogen for breast cancer and a p53 mutant for lung cancer. In HCC, a number of biomarkers have been proposed to be associated with HCC prognosis. The most commonly used biomarker is the alpha-fetoprotein (AFP) protein. In addition, the transcription factor forkhead box C1 and cyclin G1, which are highly expressed in the majority of HCC tissues, are correlated with HCC metastasis by associating with snail expression and the AKT-signaling pathway (39). SAL4 protein, which is upregulated in HCC stem cells, has been used as an HCC stem marker and HCC prognostic marker (39,40). However, these biomarkers are not sufficient for interpreting the intricate prognosis of HCC clinically. These data indicate that HSF1 is upregulated in the most developed (late stage) HCC tissues. Its expression and transcriptional activity is closely correlated with HCC malignancy and invasion, which implies that HSF1 may be used as a novel biomarker in HCC prognosis.

In conclusion, the present study demonstrated that HSF1 is upregulated in HCC tissues and cell lines. Its expression levels and transcriptional activity are correlated with HCC development. Cancer stage progression is likely to be correlated with the expression of HSF1, which indicates that HSF1 may be a useful biomarker for HCC prognosis and the development of novel therapeutic strategies for its treatment.

## Figures and Tables

**Table I:** Cohort study of HSF1 protein expression in HCC tissues.

Clinical factors	Cases (n)	HSF1 expression		Positive ratio (%)	χ^2^	P-value

		Positive	Negative			
Age, years						
<55	36	23	13	63.9	0.822	0.365^a^
≥55	31	23	8	74.2		
Gender						
Male	55	37	18	67.3	0.273	0.740^b^
Female	12	9	3	75.0		
HBV						
HBsAg (+)	53	39	14	73.6	2.863	0.112^b^
HBsAg (−)	14	7	7	50.0		
AFP						
Positive	39	26	13	66.7	0.172	0.679^a^
Negative	28	20	8	71.4		
CEA						
Positive	56	41	15	73.2	3.292	0.086^b^
Negative	11	5	6	45.5		
Wrap membrane						
Intact	27	14	13	51.9	5.935	0.015^a^
Broken	40	32	8	80.0		
Portal V thrombosis						
Yes	31	24	7	77.4	2.059	0.151^a^
No	36	22	14	61.1		
Differention						
Low	26	25	1	96.2	9.762	0.002^a^
Middle	31	19	12	61.3	5.159	0.032^b^
High	10	2	8	20.0		
TNM phase						
Phase I+II	25	11	14	44.0	11.267	0.001^a^
Phase III+IV	42	35	7	83.3		

a χ^2^-test;

b P-value.

HSF1, heat shock factor 1; HCC, hepatocellular carcinoma; portal V; portal vein; TNM, tumor, nodes and metastasis; HBV, hepatitis B virus; AFP, alpha-fetoprotein; CEA, carcinoembryonic antigen.

**Table II:** Correlation between HSF1 and Hsp70.

	Hsp70		
		
HSF1	+	−	Total
+	22^a^	3	25
−	5	10	15
Total	27	13	

a Spearman test, P<0.05.

HSF1, heat shock factor 1; HSP70, heat shock protein 70.
